# G. W. Parmly, Dentist

**Published:** 1848-04

**Authors:** 


***G. W. Parmly, Dentist.***—We are gratified to learn, from the following, translated from a foreign paper, that the professional skill and moral worth of G. W. Parmly, son of Dr. L. S. Parmly of New Orleans, is so highly appreciated abroad as to have procured for him so complimentary a distinction.—*Balt. Ed.*

"We learn that Mr. Parmly, an American surgeon dentist, has been appointed, with the royal approbation, and on the nomination of Prince Alexander of Holland, dentist to His Royal Highness.

The large number of persons who have had occasion to avail themselves of Mr. Parmly's services will be glad to learn that this well merited distinction will make it necessary for him to remain permanently in this city, where he has become so much esteemed both for his surgical talents and his professional.

Dental surgery seems to be hereditary in the Parmly family. The American papers testify that members of this family hold the first rank among the professors of this branch of surgery, in the principal cities of the Union."

